# A rare case of intussusception leading to the diagnosis of acquired immune deficiency syndrome: a case report

**DOI:** 10.1186/1752-1947-3-61

**Published:** 2009-02-11

**Authors:** Ioannis Kehagias, Stavros N Karamanakos, Spyros Panagiotopoulos, Sofia Giali, Charalambos A Gogos, Fotis Kalfarentzos

**Affiliations:** 1 Department of Surgery, School of Medicine, University of Patras, Rion University Hospital, 26500, Patras, Greece; 2 Department of Internal Medicine, School of Medicine, University of Patras, Rion University Hospital, 26500, Patras, Greece

## Abstract

**Introduction:**

Although a common cause of intestinal obstruction in children, intussusception is a rare event in the adult population living in temperate regions. It has long been known that various acquired immune deficiency syndrome related conditions of the bowel such as lymphoma, lymphoid hyperplasia, cytomegalovirus colitis and Kaposi's sarcoma can lead to intussusception. The diagnosis is particularly difficult in this population of patients due to the non-specific nature of the symptoms as well as the depressed immune response obscuring inflammation or ischemia. Though the reported acquired immune deficiency syndrome associated cases of intussusception refer to patients with known human immunodeficiency virus infection, in our case we present an intestinal intussusception as the first manifestation of human immunodeficiency virus infection.

**Case presentation:**

A 58-year-old white heterosexual Greek man with a clean medical record and no history of abdominal operation presented to the emergency department with symptoms and signs of bowel obstruction. Plain abdominal radiographs were highly suspicious for intussusception which was eventually confirmed on a computed tomography scan. Due to the patients clean medical record as well as the radiologic diagnosis of intussusception, we promptly undertook further serologic tests for human immunodeficiency virus and eventually established the diagnosis of acquired immune deficiency syndrome. The patient was operated 3 days later and this confirmed the diagnosis of small-bowel invagination due to a 4 cm polypoid growing intraluminal tumor, the pathologic examination of which revealed a diffuse high-grade B cell lymphoblastic lymphoma.

**Conclusion:**

Human immunodeficiency virus infection may have a silent course and gastrointestinal manifestations of the disease leading to intussusception might be the first clinical sign. Patients with intestinal intussusception, and the presence of risk factors for human immunodeficiency virus infection should be eligible for serologic tests for human immunodeficiency virus infection.

## Introduction

Intussusception comes from the Latin *intussuscipere *which means to take in and refers to a bowel that invaginates upon itself. Though intussusception is a common cause of intestinal obstruction in the pediatric population, it is quite uncommon in adults living in temperate regions, representing fewer than 10% of total causes [1]. Unlike childhood intussusception, which is idiopathic in 90% of cases, adult intussusception has a demonstrable cause in over 90% of cases [2].

An intraluminal tumor, submucosal edema or any process that causes dysrhythmic contractions may initiate intussusception. Colonic intussusception is most commonly caused by a primary carcinoma and benign tumors, including submucosal masses and accounts for the majority of cases of intestinal intussusception [3].

There is growing evidence from the literature associating intussusception with human immunodeficiency virus (HIV) infection [1,3-8]. Gastrointestinal manifestations of acquired immune deficiency syndrome (AIDS) that may potentially initiate an intussusception include lymphoma, lymphoid hyperplasia, cytomegalovirus (CMV) colitis and Kaposi's sarcoma [9].

We present a case of intestinal intussusception as the first manifestation of HIV infection in a middle-aged man.

## Case presentation

A 58-year-old, white heterosexual Greek man with a clean medical record and no history of abdominal operation presented to the emergency department with a 2-week history of gradually worsening abdominal pain. Though the patient had been experiencing flatus daily, he reported no bowel movements over the last 5 days. Furthermore, the patient had worsening nausea and vomiting as well as abdominal distention leading to inability to tolerate oral intake.

Physical examination revealed a well-nourished, mildly febrile patient (37.5°C). He was hemodynamically stable and his abdomen, though soft, was distended and tender in the hypogastrium and right lower quadrant. No hernia was apparent. Bowel sounds were scarce and rectal examination showed heme-positive stools. Laboratory tests revealed a peripheral leukocyte count of 4080/μl with a normal differential count and a hematocrit of 30%. Electrolytes, liver biochemistry and amylase levels were normal.

**Figure 1:**
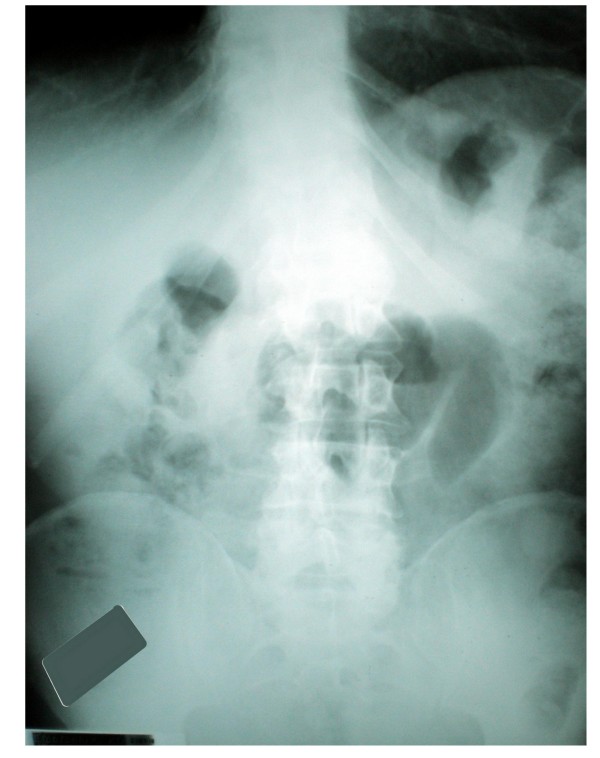
**Plain abdominal radiograph showing dilated loops of small bowel in the right hemiabdomen and a soft tissue mass**.

**Figure 2:**
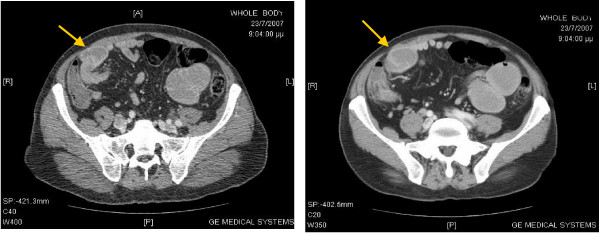
**Contrast enhanced abdominal tomography at the level of the umbilicus showing a characteristic 'target mass' (arrows) in the right abdomen**.

Plain abdominal radiographs showed multiple air-fluid levels in distended small-bowel loops and air in the colon indicating partial small bowel obstruction (Figure 1). A computed tomography (CT) scan of the abdomen revealed dilated loops of the small intestine and a transition point to decompressed loops at the level of the mid-ileum, as well as a typical 'target sign' of intussusception (Figure 2).

Though serologic tests for HIV infection are not routinely performed in our department for patients with intestinal obstruction, it was our awareness of the association of intussusception with various AIDS-related conditions of the bowel, as well as the patient's clean medical and surgical records that made further screening necessary. Surprisingly, the patient was seropositive for HIV infection and had a cluster of differentiation 4 (CD4) cell count of 274/μl and viral load of 129,000 copies/ml.

**Figure 3:**
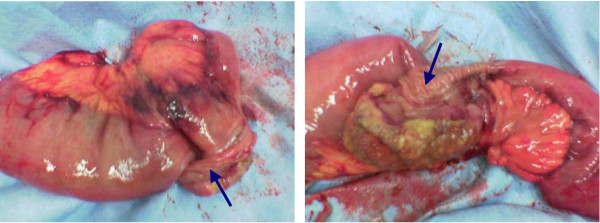
**Resected small bowel segment showing an intraluminal growing mass (arrows) as the underlying reason for the intussusception**. Pathologic examination of the specimen revealed a diffuse high-grade B cell lymphoblastic lymphoma.

Laparotomy was performed 3 days later only to confirm the diagnosis of small-bowel invagination due to a 4 cm polypoid growing intraluminal tumor (Figure 3). Pathologic examination of the specimen revealed a diffuse high-grade B cell lymphoblastic lymphoma. The patient had an uneventful recovery and was discharged from hospital on the 6^th ^postoperative day.

## Discussion

Bowel obstruction is one of the most common complaints driving patients to our emergency department. In the vast majority of cases, a history of previous abdominal operation is revealed making adhesions the leading cause of intestinal obstruction. Other less common causes of intestinal obstruction include incarcerated hernias, malignant disease and inflammatory bowel disease. In cases of intestinal obstruction where the above pathologic conditions are not revealed, it is a real challenge for the surgeon to undertake the diagnosis.

Though a common cause of intestinal obstruction in children, intussusception is a rare event in the adult population living in temperate regions, accounting for only 2 to 3 cases per 1 million population reported annually [1].

It has long been known that various AIDS-related conditions of the bowel can lead to intussusception [10,11]. Nonetheless, the diagnosis is particularly difficult in this population of patients due to the non-specific nature of the symptoms as well as the depressed immune response leading to low leukocyte count and thus obscuring any inflammation or ischemia [6].

Contrast-enhanced CT of the abdomen is the diagnostic tool of choice. Intussusception has a pathognomonic appearance on CT scan, the 'target sign', with a visible appearance of an outer bowel wall circumscribing the inner wall. Additionally, a hypodense area which represents invaginated mesenteric fat is often apparent within the intussusceptum.

Intussusception appears to be more common in HIV infected patients due to the increased incidence of pathologic small bowel processes [12,13]. The interesting feature of our case is that our patient did not have a documented HIV infection. Instead, it was his clean medical record as well as the radiologic diagnosis of intussusception that prompted us to undertake further serologic tests and eventually to establish the diagnosis.

We are aware of cases of intussusception in HIV patients reported elsewhere in the literature [1,4-6,8,9]. However, we believe that this is a rare case of silent HIV infection diagnosed via a gastrointestinal manifestation of the disease.

## Conclusion

Though a rare cause of intestinal obstruction in adults, intussusception has been shown to have a significant correlation with HIV infection because of its association with a variety of infective and neoplastic conditions of the bowel. Apparently, HIV infection may have a silent course and gastrointestinal manifestations of the disease leading to intussusception might be the first clinical sign. Therefore, patients with intestinal intussusception, and the presence of risk factors for HIV infection, should be eligible for serologic tests for HIV infection. In these patients, surgical reduction in the intussusception is well tolerated and is of clear benefit.

## Abbreviations

HIV: human immunodeficiency virus; AIDS: acquired immune deficiency syndrome; CMV: cytomegalovirus; CD4: cluster of differentiation 4; CT: computed tomography.

## Consent

Written informed consent was obtained from the patient for publication of this case report and any accompanying images. A copy of the written consent is available for review by the Editor-in-Chief of this journal.

## Competing interests

The authors declare that they have no competing interests.

## Authors' contributions

IK was the major contributor in the conception and design of the study as well as the completion of the operation. SNK and SP collected the data, wrote the paper and were assistants in the operation. SG made substantial contributions to the acquisition and analysis of data, was the attentant physician both during hospitalization and in the follow up visits and CAG was responsible for treatment decisions concerning the patient and he revised the manuscript for important intellectual content, FK gave final approval of the version to be published. Finally, all authors read and approved the final manuscript.

## References

[B1] Blazes DL Lipscomb SJ Schoenfeld PS Martin GJ Intussusception in an HIV infected patient: A case report and review of the literature AIDS Read 2001 11 525 528 11708085

[B2] Agha FP Intussusception in adults AJR 1986 146 527 531 3484870 10.2214/ajr.146.3.527

[B3] Silverman PM Hayes WS Cooper CJ Fanney D West MS Forer L Hartman DS Davidson AJ Stull MA Abdominal case of the day AJR 1990 154 1325 13302110750

[B4] Balthazar EJ Reich CB Pachter HL The significance of small bowel intussusception in Acquired Immune Deficiency Syndrome Am J Gastroenterol 1986 81 1073 1075 3776957

[B5] Meyerson S Desai TK Polidori G Raval MF Ehrinpreis MN A case of intussusception and lymphoid hyperplasia in a patient with AIDS Am J Gastroenterol 1993 88 303 306 8424440

[B6] Visvanathan R Nichols TT Reznek RH Acquired immune deficiency syndrome-related intussusception in adults Br J Surg 1997 84 1539 1540 10.1002/bjs.1800841112 9393273

[B7] Wetter A Schaudt A Lehnert T Schmidt-Matthiesen A Jacobi V Vogl TJ Small-bowel intussusception as a rare differential diagnosis in HIV-positive patients with acute abdomen Eur Radiol 2006 16 952 953 10.1007/s00330-005-2785-y 15895234

[B8] Farrier J Dinerman C Hoyt DB Coimbra R Intestinal lymphoma causing intussusception in HIV+ patient: A rare presentation Curr Surg 2004 61 386 389 10.1016/j.cursur.2004.01.010 15276346

[B9] Hofstetter SR Stollman N Adult intussusception in association with acquired immune deficiency syndrome and intestinal kaposi's sarcoma Am J Gastroenterol 1986 83 1304 1305 3189268

[B10] Wood BJ Kumar PN Cooper C Silverman PM Zeman RK AIDS-associated intussusception in young adults J Clin Gastroenterol 1995 21 158 162 10.1097/00004836-199509000-00019 8583084

[B11] Wilson SE Robinson G Williams RA Stabile BE Cone L Sarfeh IJ Miller DR Passaro E Jr Acquired immune deficiency syndrome (AIDS). Indications for abdominal surgery, pathology, and outcome Ann Surg 1989 210 428 434 10.1097/00000658-198910000-00002 2552944 PMC1357915

[B12] Chambers AJ Lord RSA Incidence of acquired immune deficiency syndrome (AIDS)-related disorders at Laparotomy Br J Surg 2001 88 294 297 10.1046/j.1365-2168.2001.01654.x 11167884

[B13] Clayton F Clayton C Gastrointestinal pathology in HIV-infected patients Gastroenterol Clin 1997 26 191 240 10.1016/S0889-8553(05)70293-4 9187923

